# Supplementary material for: Lossless enrichment of trace analytes in levitating droplets for multiphase and multiplex detection
Source: Nat Commun. 2022 Dec 17;13:7807. doi: 10.1038/s41467-022-35495-9 (PMC9759559; doi:10.1038/s41467-022-35495-9)
Supplement: Supplementary file 1 — Supplementary Information File [file 41467_2022_35495_MOESM1_ESM.pdf]

Supplementary Information for

Lossless enrichment of trace analytes in levitating droplets for multiphase and multiplex detection

Xueyan Chen[1,2§], Qianqian Ding[1,§], Chao Bi[3], Jian Ruan[2*], and Shikuan Yang[1,2,4,5*]

[1]Institute for Composites Science Innovation, School of Materials Science and Engineering, Zhejiang University, Hangzhou 310027, China

[2]Department of Medical Oncology, The First Affiliated Hospital, Zhejiang University School of Medicine, Hangzhou 310003, China

[3]Core Facilities, Zhejiang University School of Medicine, Hangzhou 310003, China

[4]State Key Laboratory of Fluid Power and Mechatronic Systems, Zhejiang University, Hangzhou 310027, China

[5]Baotou Research Institute of Rare Earths, Baotou 014030, China

[§]These authors contributed equally to this work.

*Corresponding author. Email: software233@zju.edu.cn; shkyang@zju.edu.cn

Supplementary Figures

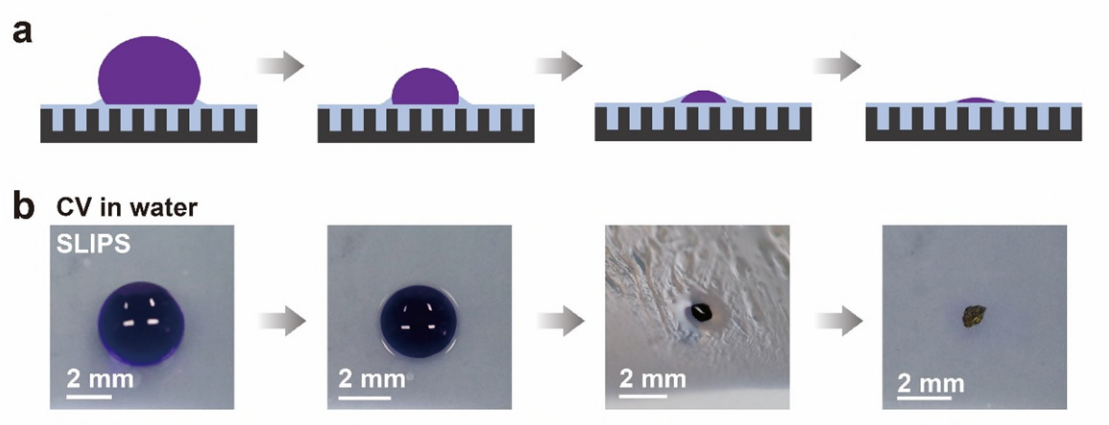

Supplementary Figure 1. The lubricant oil within the SLIPSs tended to wrap the evaporating droplet, influencing the SERS performance. (**a**) Schematic of the analyte enrichment process on slippery liquid-infused porous surfaces (SLIPSs) during solvent evaporation. (**b**) Enrichment of CV molecules from their aqueous solutions at 1 mM concentration on SLIPSs. The “coffee ring” effect was avoided, while the lubricant oil wrapped the eventual aggregate.

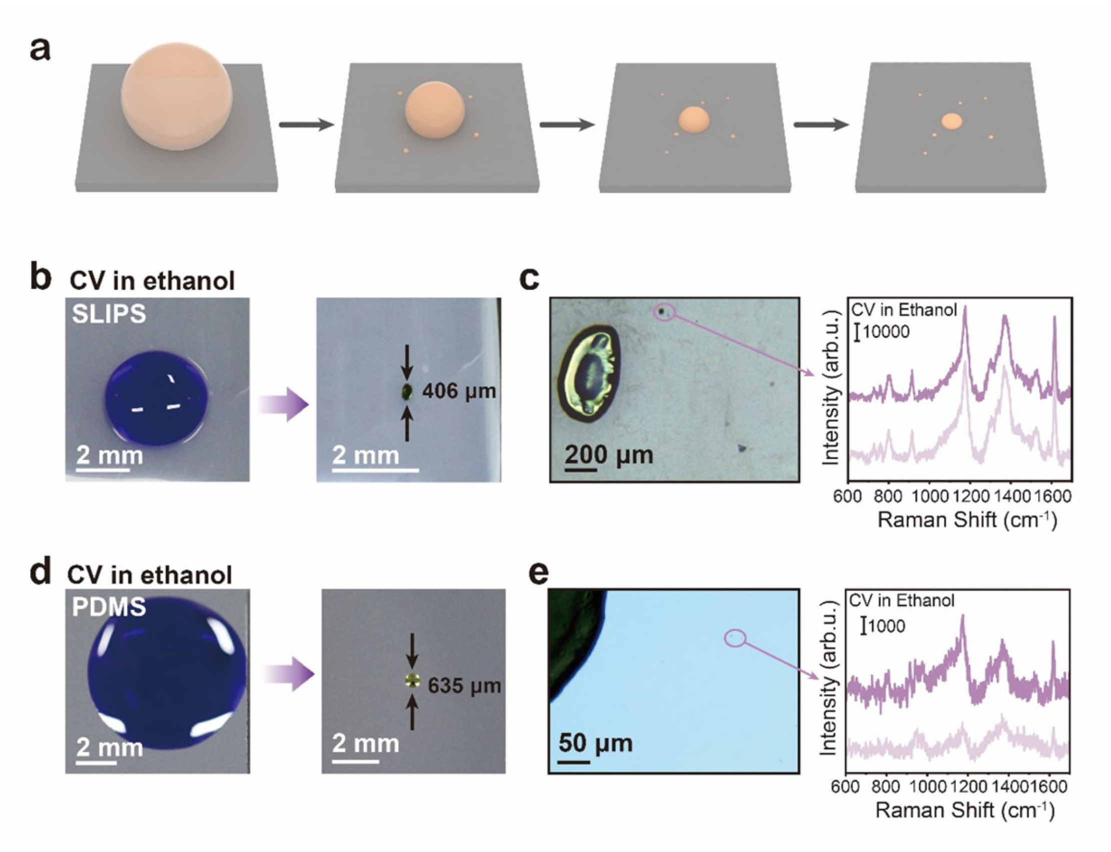

Supplementary Figure 2. Analyte loss during solvent evaporation induced analyte enrichment on slippery surfaces. (**a**) Schematic of the analyte loss on solid SERS platforms during solvent evaporation. (**b**) An analyte aggregate was formed on SLIPSs after solvent evaporation. (**c**) Tiny analyte aggregates formed after evaporating CV ethanol solutions at 1 mM concentration on the SLIPSs under microscope observations. Raman spectra proved that the aggregates were CV molecules. (**d**) Aggregates formed after evaporating CV ethanol solutions at 1 mM concentration on the slippery polydimethylsiloxane (PDMS) brush surface. (**e**) Tiny aggregates formed under microscope observations. Raman spectra proved that the aggregates were CV molecules.

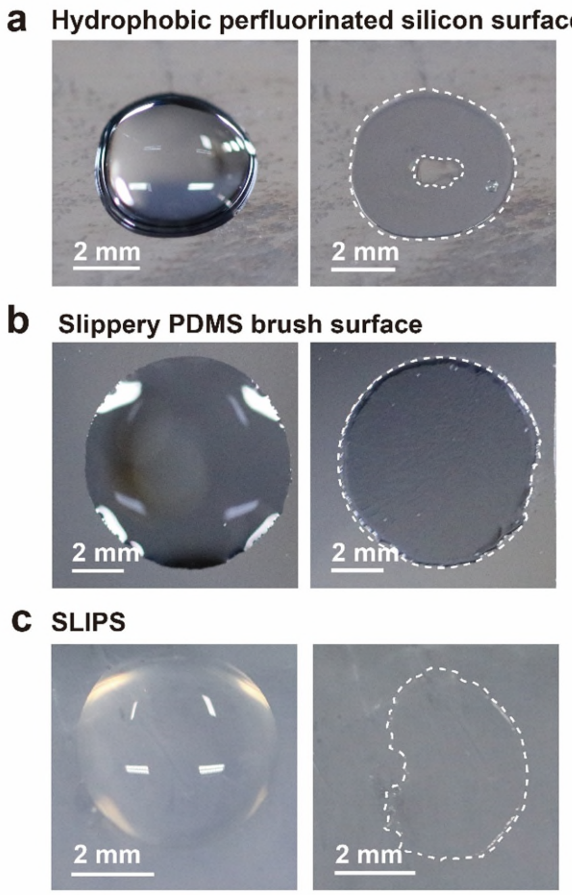

Supplementary Figure 3. No enrichment could be achieved during solvent evaporation for sticky HPC ethanol solutions (1 wt.%) on any solid surfaces. (**a**) Hydrophobic perfluorinated silicon surface. (**b**) Slippery PDMS brush surface. (**c**) SLIPS.

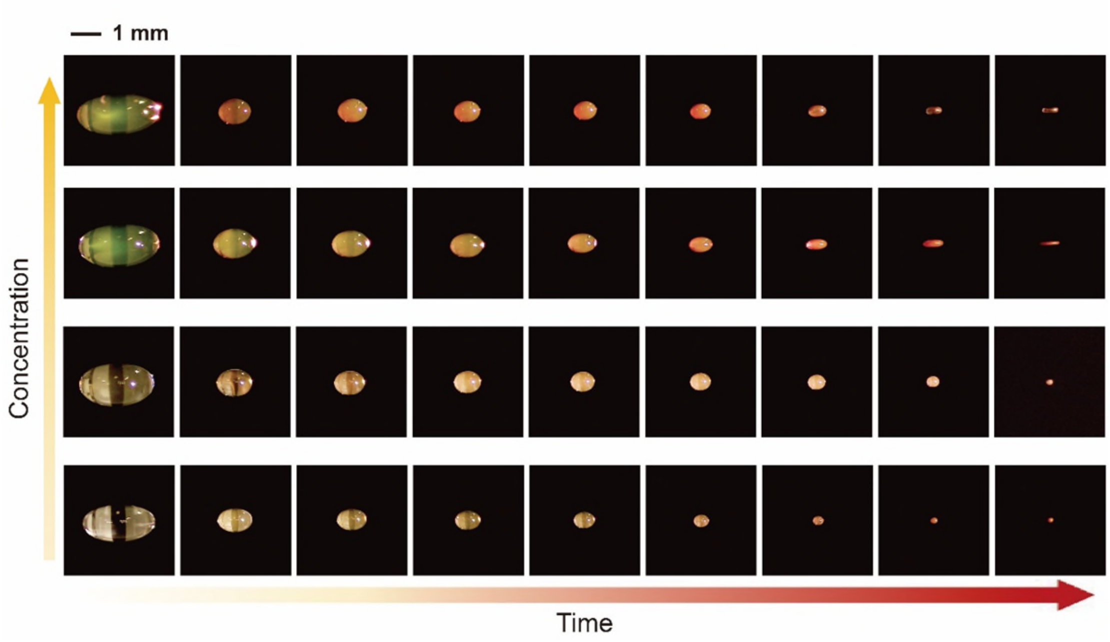

Supplementary Figure 4. Enrichment process of 10 μL of Au nanosphere colloids with concentrations ranging from 0.4 nM to 40 pM using the DLE platform. From top to bottom: 0.4 nM, 0.2 nM, 80 pM, and 40 pM.

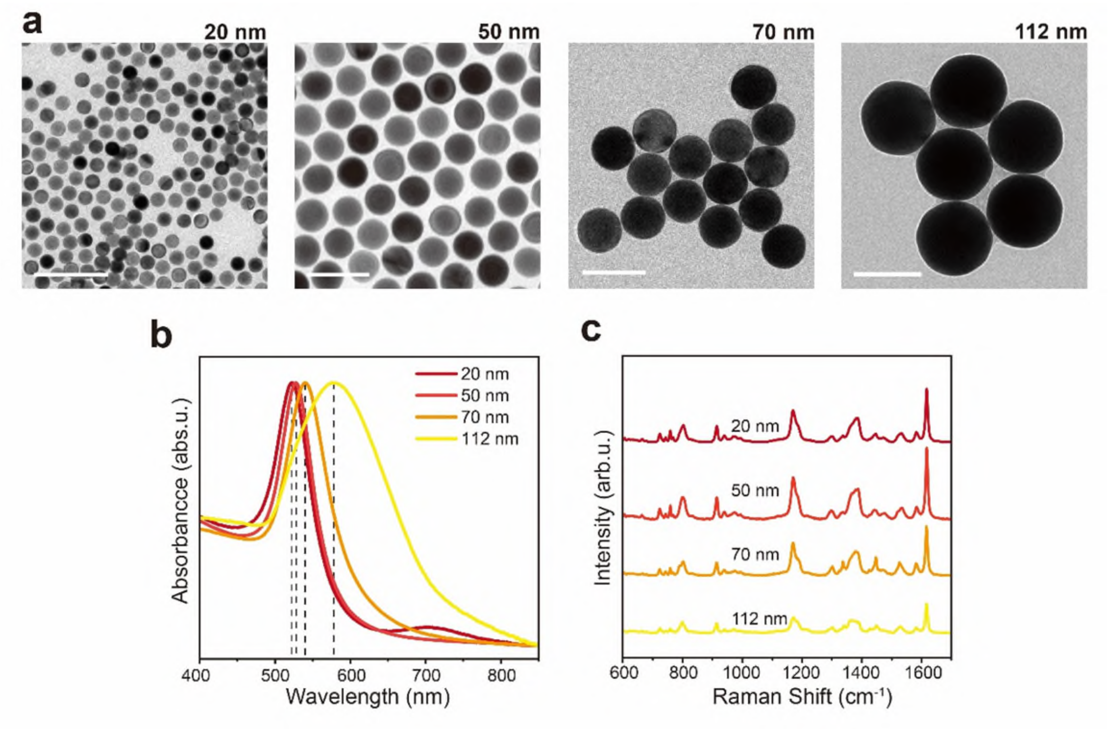

Supplementary Figure 5. SERS performance of Au nanospheres of different sizes. (**a**) Au nanospheres with different diameters. Scale bar: 100 nm. (**b**) UV-Vis absorbance spectra of Au nanospheres with different sizes. (**c**) SERS spectra of CV molecules enriched from 10 μL of their ethanol solutions containing 10 nM CV molecules and 40 pM of different sized Au nanospheres using the DLE platform.

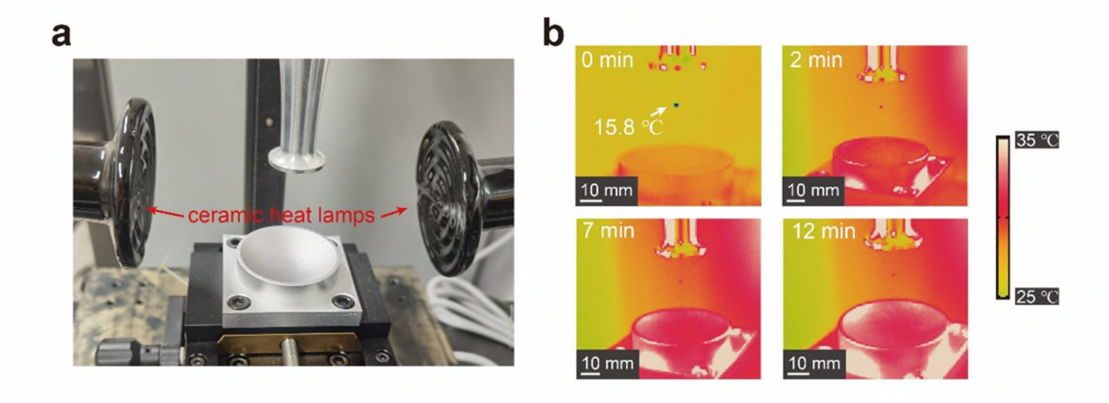

Supplementary Figure 6. The heating lamps increased the temperature of the levitating droplet and accelerated the solvent evaporation process. (**a**) Photograph of two ceramic heating lamps (Power: ~ 100 W; diameter: ~ 72 mm). (**b**) The temperature of the 10 μL of 0.4 nM Au nanosphere colloids increased from 15.8 °C to 30.3 °C in two minutes under the heating lamp irradiation. The air temperature around the droplet was ~ 55 °C. Room temperature: 22 °C. 10 μL of ethanol/water droplet needs ~ 2 h to evaporate (temperature: ~ 22 °C; humidity: ~ 55%). Under the heating lamps, the evaporation time of the ethanol and the water droplet was 28 min and 33 min, respectively. When the ambient temperature was higher and the humidity was lower, for example, 35 °C and 40%, the evaporation time was further reduced to 18 min for the ethanol droplet and 27 min for the water droplet.

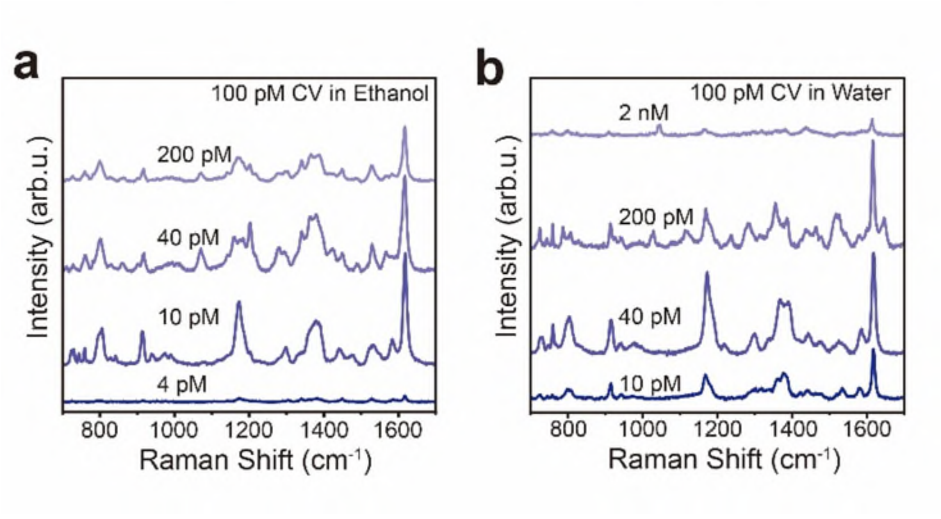

Supplementary Figure 7. The optimum amount of Au nanospheres to get the highest SERS enhancement. (**a** and **b**) SERS spectra of CV molecules enriched from 100 pM ethanol and water solutions with different amounts of Au nanospheres using the DLE platform, respectively.

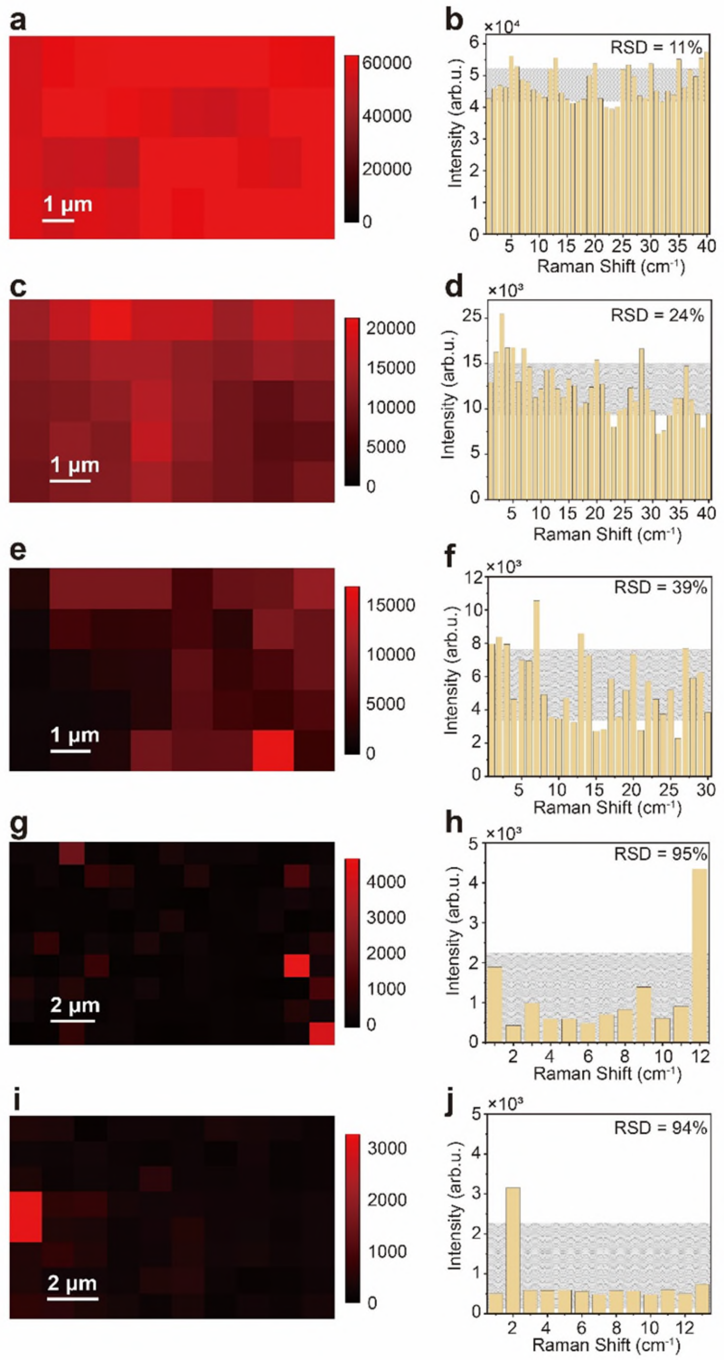

Supplementary Figure 8. SERS mapping results of the 1616 cm^{-1} SERS peak of the CV molecules enriched from different concentrations of their ethanol solutions by the DLE platform. (**a**), (**c**), (**e**), (**g**), and (**i**): 10 nM, 100 pM, 1 pM, 10 fM, and 100 aM, respectively. (**b**), (**d**), (**f**), (**h**), and (**j**): The corresponding relative standard deviation.

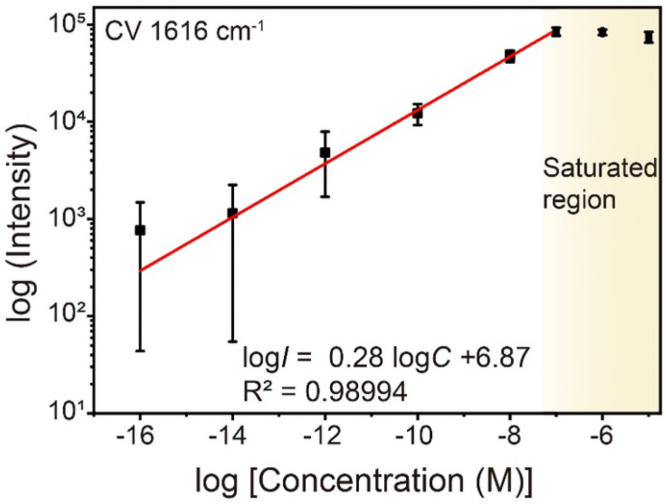

Supplementary Figure 9. The saturation concentration for the DLE-SERS sensing platform. SERS intensity at 1616 cm^{-1} as a function of the concentration of the CV ethanol solutions. When the concentration of the CV molecules was larger than ~ 100 nM, the SERS intensity did not increase as the concentration was further increased (shaded region). The error bars were obtained based on more than 10 spectra.

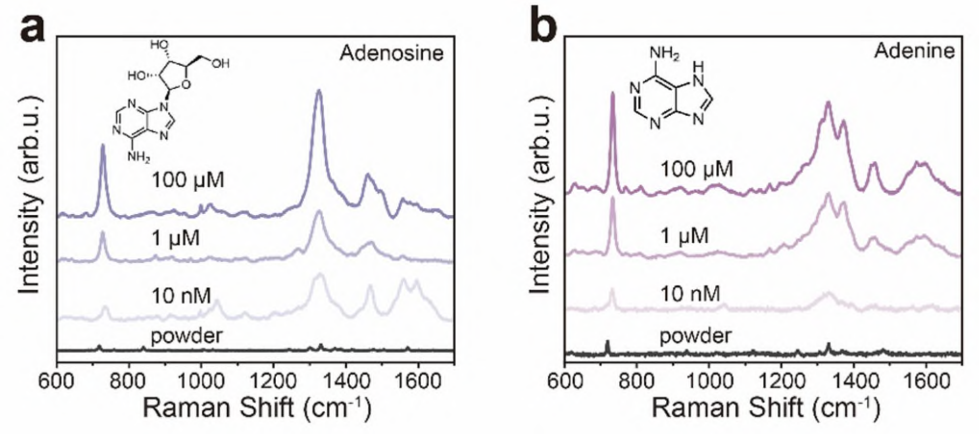

Supplementary Figure 10. SERS detection of the biomolecules by the DLE platform. (**a**) SERS spectra of adenosine at different concentrations. (**b**) SERS spectra of adenine at different concentrations.

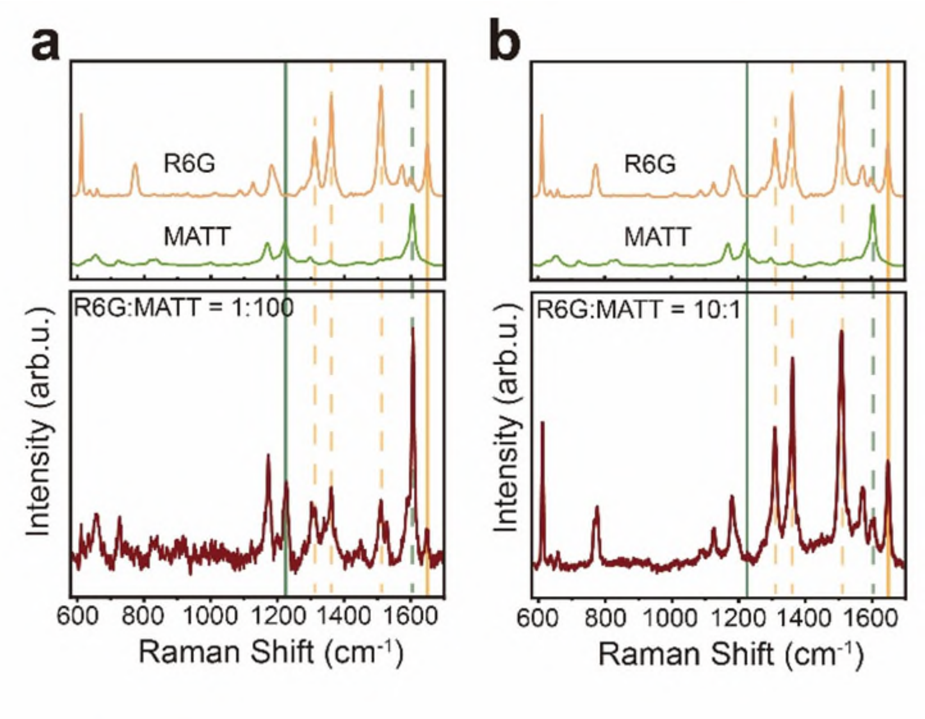

Supplementary Figure 11. Multiplex SERS detection by the DLE platform. (**a**) SERS spectra of R6G (500 pM) and MATT (50 nM) molecules after enrichment from 10 μl of their ethanol solutions using the DLE platform. (**b**) SERS spectra of R6G (50 nM) and MATT (5 nM) molecules after enrichment from 10 μl of their ethanol solutions using the DLE platform.

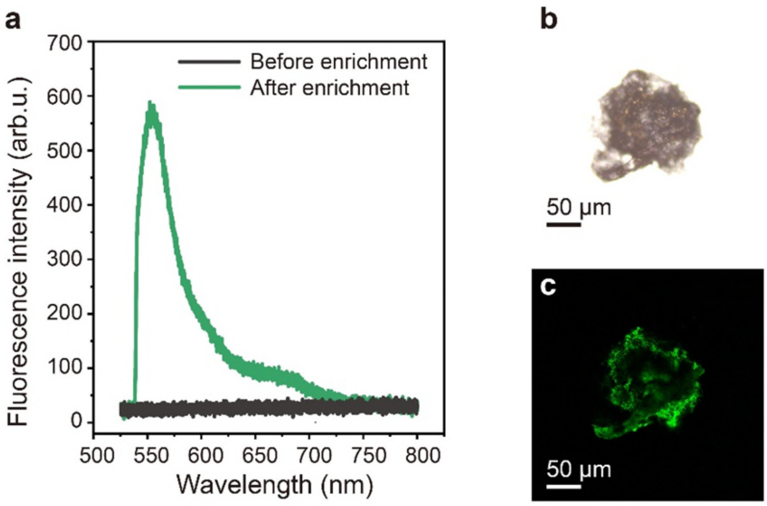

Supplementary Figure 12. The photoluminescence enhancement of R6G molecules using the DLE platform. (**a**) Fluorescence spectra of 1 fM R6G ethanol solutions before and after the DLE process. The excitation and the emission wavelength of R6G were 532 nm and 555 nm, respectively. (**b**) Microscope image of the R6G aggregate. (c) Fluorescence image of the R6G aggregate after the DLE process excited by 488 nm light.
