# Supplementary material for: Lossless enrichment of trace analytes in levitating droplets for multiphase and multiplex detection
Source: Nat Commun. 2022 Dec 17;13:7807. doi: 10.1038/s41467-022-35495-9 (PMC9759559; doi:10.1038/s41467-022-35495-9)
Supplement: Supplementary file 3 — Description of Additional Supplementary Information [file 41467_2022_35495_MOESM3_ESM.pdf]

Description of Additional Supplementary Information File

Title: Supplementary Movie 1

Description: Aqueous solutions introduced into toluene to achieve multiphase analyte enrichment.

Title: Supplementary Movie 2

Description: Crystal violet molecules enriched into a ~100 µm violet droplet after solvent evaporation.

Title: Supplementary Movie 3

Description: 500 nm polystyrene spheres dispersed in ethanol assembled into a big ball after ethanol evaporation.
